# Advancing Optimal Development in Children: Examining the Construct Validity of a Parent Reflective Functioning Questionnaire

**DOI:** 10.2196/11561

**Published:** 2019-05-09

**Authors:** Monica De Roo, Gina Wong, Gwen R Rempel, Shawn N Fraser

**Affiliations:** 1 Graduate Centre for Applied Psychology Faculty of Health Disciplines Athabasca University Athabasca, AB Canada; 2 Centre for Nursing and Health Studies Faculty of Health Disciplines Athabasca University Athabasca, AB Canada

**Keywords:** mentalization, parent reflective functioning, questionnaire design, parenting

## Abstract

**Background:**

Parental reflective functioning (PRF) is the capacity parents have to understand their own mental states and those of their children, as well as the influence of those mental states on behavior. Parents with greater capacity for PRF are more likely to foster secure attachment with their children. The Parental Development Interview is a gold standard measure of PRF but is hampered by cost, training, and length of administration. The 18-item Parent Reflective Functioning Questionnaire (PRFQ-18) is a simpler option developed to capture 3 types of PRF: (1) prementalizing, (2) parent’s certainty, and (3) interest and curiosity surrounding a child’s mental state.

**Objective:**

The aim of this study was to examine the factor structure and select psychometric properties of the PRFQ in a sample of Canadian parents.

**Methods:**

We examined the factor structure and discriminant and construct validity of the PRFQ-18 among 306 parents (males=120 and females=186) across Canada; the age range of children was 0 to 12 years. Parents also completed Web-based measures of perceived stress, parental coping, parenting competence, and social support.

**Results:**

A confirmatory factor analysis confirmed the hypothesized 3-factor structure of the PRFQ-18 providing evidence that the PRFQ-18 may be a useful and practical measure of PRF in Canadian adults and showed minor revisions may improve the suitability of the PRFQ-18 for assessing PRF.

**Conclusions:**

These results add support for the construct validity of the PRFQ-18.

## Introduction

### Background

Decades of research unequivocally demonstrate that reflective processes enhance parental insight and sensitivity toward children’s emotions [1-5]. Reflective functioning (RF), first introduced by Fonagy et al in 1991, describes an individual’s cognitive capacity to recognize and interpret one’s own mental state as well as the mental state of others to identify and comprehend the meaning behind behavior [2,6]. In the literature, RF is akin to mentalizing, which is a fundamental and intrinsic human capacity to regulate affect and attune to interpersonal relationships [7]. Similarly, maternal mind-mindedness is described as a mother’s ability to recognize her child as a separate agent with independent thoughts, experiences, and emotions [8], and parental insightfulness is defined as a parent’s representation of their child’s intentions and mental states [3].

Rather than a natural ability, RF is believed to develop through the internal organization of an individual’s understanding of one’s own and other’s feelings and behaviors through experiences, social and emotional information, meaning making [2,6], and interactions with primary caregivers [2,6,9]. In addition, social interactions, family structure, family size, parenting quality [9], and environmental responses [10] influence the development of RF. The development of the RF neurological function is noteworthy as it provides individuals with the ability to predict behavior, distinguish between manifestation and reality, and enhance interpersonal communications, self-organization [6], and impulse control and affect regulation [9]. Understanding the development of RF is crucial, given the relationship between how a parent chooses to respond to their child’s needs, which affects the child’s attachment status [2,4,11] as well as the child’s development and capacity to mentalize [1], and circumscribes the health of the parent-child relationship overall [5,12].

Parental reflective functioning (PRF) pertains to a parent’s proclivity to understand and comprehend mental states influencing their child’s behavior [13], playing a crucial role in how parents respond to their child’s needs and feelings [4,14] and behaviors [6]. PRF is a core cognitive capacity that connects parents to their child’s emotions and also to their own early attachment experiences in an integrated way to see their parents’ experience as distinct from their own and to provide a secure base for their own children [1,6,7].

PRF is described as an attachment-related concept [15], wherein parental ability to interpret a child’s mental state and the parents’ responsiveness, or lack thereof, act as a conduit that establishes the attachment status of children. In other words, a child’s attachment status is emblematic of the parent’s capacity for PRF. The interrelationships between attachment and (1) child psychopathology; (2) inflammation and health; (3) neurobiology; (4) empathy, compassion, and altruism; and (5) school readiness have been substantiated [16]. In addition, the quality of attachment to a parent has been shown to be predictive of numerous developmental outcomes in children such as general well-being, self-esteem, social competence with peers, problem-solving abilities, academic success, behavioral outcomes, and resilience [17-21].

Parents with high RF who possess clear mentalizing abilities have a positive influence on the biological, interpersonal, cognitive, and emotional experiences of the child [1]. They understand their own mental states and those of their children, as well as the influence of those mental states on behavior [1]. In addition, those with high RF are better able to perceive themselves as parents and their relationships with their child, therefore seeking out social support [22] and enhancing parental coping abilities [23].

Initial purposes of PRF measures were to directly measure maternal representations of their child [24] and the PRF functions that influence the intergenerational transmission of attachment [1,4]. The application of PRF measures has expanded to different contexts such as drug use disorders [25], mothers with childhood maltreatment [26,27], infant distress [28], and parenting programs [12,29-33].

The gold standard narrative-based RF measure assessing parental representations of their relationship with their child is the Parent Development Interview (PDI) [24]. The interview takes approximately 2 hours to administer and is audio recorded for transcribing. The original 45-item PDI scoring process involved a trained coder who utilized narrative data to evaluate RF across 4 factors: (1) awareness of the nature of mental states, (2) the ability to tease out underlying behavior of mental states, (3) identifying developmental aspects of mental states, and (4) the mental states in relation to the interviewer [24]. The PDI has been revised to a 40-item and 29-item measure to assess parent RF relative to their own child, their parents, and self (personal communication, A. Slade, January 2016). Trained coders rate each item on a Likert scale from −1 to 9 to produce an RF score [24]. A limitation in terms of application and use of the PDI is that the training and coding requirements for the PDI are time consuming and expensive. Clinicians and researchers may experience these prohibitive factors, making the PDI unattractive and unrealistic.

As an alternative to the narrative-based PDI, the Parent Reflective Functioning Questionnaire (PRFQ-18) is an 18-item self-report measure [13] which examines 3 domains of PRF: (1) prementalizing modes (PM), designed to capture a parent’s inability to hold the child’s mental state in mind; (b) interest and curiosity in mental states (IC), intended to capture the level of interest in parents thinking about their child’s mental states; and (c) certainty about mental states (CMS), measuring a parent’s acknowledgment that their thoughts about their child’s mental states are accurate [13]. The main advantage of the PFRQ-18 is that it is a brief screening tool of RF designed to meet the growing demand of measures to assess the effectiveness of interventions to improve parent-child attachment and PRF [13].

The PRFQ-18 is an open source questionnaire available [34]. There is no training requirement; the questionnaire takes about 10 min to complete and the scoring syntax is downloadable. The questionnaire can be completed on paper or Web-based and is available in 10 languages. Parents rate each subscale item on a Likert scale from 1 (*strongly disagree*) to 7 (*strongly agree*). The preliminary studies 1 and 2 [13] have provided evidence supporting the validity and reliability of the PRFQ-18 for measuring PRF.

In Study 1, PRF was examined in mothers with children aged 0 to 36 months [13]. Construct validity of the PRFQ-18 was supported with a confirmatory factor analysis (CFA) of a 3-factor model with a good fit (χ^2^_123_=217.73; *P*<.001; χ^2^/df=1.77; root mean square error of approximation [RMSEA]=.05; CI 0.04 to 0.06; comparative fit index [CFI]=.91; non-normed fit index [NNFI]=.91). Internal consistencies were good for PM (.70), IC (.82), and CMS (.75), and discriminant validity was identified in negative correlations between the PRFQ-18 factors and demographic features. As expected, PM was positively correlated to attachment avoidance, attachment anxiety, and symptomatic distress, whereas IC and CMS were not.

In Study 2, opposing results in the correlations between IC and CMS were found [13], revealing factorial variance across mothers and fathers. In contrast to Study 1, correlations between PRFQ-18 factors and demographic features were nonexistent or moderately related to the subscales; no relationships were found between fathers PM, attachment, and symptomatic distress, indicating differences in factor loading among mothers and fathers. On the contrary, Study 2 identified similar correlations between parenting stress and mothers and fathers. Parenting stress had negative correlations with IC and CMS but positive correlations with PM on all subscales including parental competence. Furthermore, in Study 3, the PRFQ-18 was utilized with the Strange Situation Procedure (SSP) [35]. Preliminary studies found correlations between PM and IC and the SSP [13], supporting the notion that a parent’s ability to hold their child’s mental state is related to attachment security. Given that RF has previously been found to significantly correlate with infant attachment [2], Study 3 strongly supports the validity of the PRFQ-18 as an indicator of PRF.

These studies from the originators of the PRFQ-18 provide some initial information on the psychometric properties of the PRFQ-18, but further research is warranted to offer an independent examination of the construct validity of the PRFQ-18. The purpose of this study was to examine the construct validity of the PRFQ-18 in a diverse sample of Canadian parents.

### Hypotheses

On the basis of results of existing studies of the PRFQ-18 and existing conceptualizations for PRF, we tested 6 hypotheses:

1. A CFA will support a 3-factor structure in the PRFQ-18, illustrating 3 distinct characteristics of RF (PM, IC, and CMS).
2. Small-to-moderate correlations will exist between the 3 PRFQ-18 subscales.
3. PM will negatively correlate with parental competence; IC and CMS will positively correlate with parental competence.
4. PM will positively correlate with parenting stress; IC and CMS will negatively correlate with parenting stress.
5. PM will negatively correlate with social support; IC and CMS will positively correlate with social support.
6. PM will negatively correlate with parental coping; IC and CMS will positively correlate with parental coping.
7. The PFRQ-18 will demonstrate measurement invariance between men and women.

## Methods

### Ethical Approval

Ethical approval for this study was obtained through our university’s Research Ethics Board. Subjects were then randomly contacted through SurveyMonkey’s *Survey Audience* to participate in a study of *Experiences in Parenting*. The survey panel members were randomly selected, contacted, and screened by SurveyMonkey, and the authors had no role in the recruitment and selection process other than defining the inclusion criteria for SurveyMonkey. Eligible participants were parents of at least 1 child living in the home aged 12 years or under. After screening for eligibility, parents completed the Web-based PRFQ-18, 4 additional measures, and the demographic data form, 52 questions excluding demographic data questions. Only 1 parent from each household provided study data.

### Measures

#### Parent Reflective Functioning Questionnaire

The PRFQ-18 [13] is an 18-item self-report measure for parents with children aged 0 to 5. It measures PRF across 3 domains: (1) PM (items 1, 4, 7, 10, 13, and 16), (2) IC (items 3, 6, 9, 12, 15, and 18), and (3) CMS (items 2, 5, 8, 11, 14, and 17). Parents are required to rate each subscale item on a Likert scale from 1 (*strongly disagree*) to 7 (*strongly agree*). The questionnaire is available from the authors.

#### Parenting Sense of Competence Scale

The Parenting Sense of Competence Scale (PSOC) is a 17-item self-report measure for assessing parents’ sense of confidence and satisfaction with their parenting [36]. Owing to poor factor loading for item 17 (.40), the PSOC was revised to a 16-item measure to assess parent sense of competence across 2 factors: (1) satisfaction and (2) efficacy and had good internal consistency for the total score (.79), satisfaction (.75), and efficacy (.76) [36]. Internal consistencies were reported .80 for both efficacy and satisfaction in mothers and .77 for efficacy and .80 for satisfaction in fathers [37]. Parents are required to rate each subscale item on a Likert scale from 1 (*strongly disagree*) to 6 (*strongly agree*). Higher scores indicate greater parenting self-confidence. For our sample, satisfaction internal consistency estimates were alpha=.89 and omega=.92 and efficacy estimates were alpha=.86 and omega=.90.

#### Perceived Stress Scale

The Perceived Stress Scale (PSS) [38] is originally a self-reported 14-item measure to examine the degree to which individuals view situations as stressful. The original PSS had good reliability in 3 preliminary samples (.84, .85, and .86) and in 2 test-retest samples (.85 and .55). The PSS was later revised to a simple 4-item (items 2, 6, 7, and 14) scale with an alpha reliability coefficient of .60, identifying it to be an adequate brief measure of perceptions of stress [39]. The PSS requires individuals to rate 4 items on a Likert scale from 0 (*never*) to 4 (*very often*). For our sample, internal consistency estimates were .68 for alpha and .85 for omega.

#### Medical Outcome Study Social Support Survey

The Medical Outcome Study Social Support Survey (MOS-SSS) [40] 12-item measure has 4 social support domains (tangible support, emotional information support, affectionate support, and positive interaction) [40]. The MOS-SSS has exhibited excellent reliability (.94) and good internal consistency for tangible support (.87), emotional information support (.91), affectionate support (.88), and positive interaction (.92) [40]. The MOS-SSS requires individuals to rate each subscale item on a Likert scale from 1 (*none of the time*) to 5 (*all of the time*). For our sample, internal consistency for the subscales ranged from alpha=.91 to .93 and from omega=.94 to .98.

#### Parent Coping Scale

The Parent Coping Scale (PCS) [41] is a single item scale to assess parent’s perception of their own ability to cope with parenting. A preliminary study of the PCS found strong intraclass correlation coefficients (.93) and concurrent criterion validity (.54) with the Parenting Self-Agency Measure [42]. The PCS requires parents to respond to a single question (“How are you coping with being a parent these days?”) on a response scale ranging from 1 (*I feel I am not coping at all these days*) to 5 (*I always feel I am coping really well—things never or hardly ever get on top of me*).

### Data Analysis

Data were exported into IBM SPSS 23 for analysis. The data analysis was conducted in 3 stages. First, data were screened for outliers and missing data. Outliers were defined as unusually influential data or data with unusual or extreme values. For example, responses outside of possible ranges were considered unusual. Given the nature of the constrained response options (eg, Web-based Likert scales), we did not notice any unusual values. We also examined partial regression plots and did not notice any outliers. Screening for influential multivariate outliers was examined with Mahalanobis d-squared in IBM AMOS [43], and we noted no distinct values indicating influential outliers. Missing data were not imputed, given the small rates of missing data. Second, the hypothesized factor structure of the PRFQ-18 was tested using a CFA in IBM AMOS 23 graphics [43] and R 3.3.2 [44]. The CFA was conducted in the usual iterative fashion [45], that is, we tested the initial hypothesized factor structure as indicted in preliminary studies [13] followed by changes to the model based on model fit, nonsignificance of path coefficients, and/or substantive suggestions offered by modification indices. Model fit was determined by consulting multiple fit indices, consistent with suggested practice (eg, [46,47]). We consulted chi-square as a global fit index. Whereas this is not a particularly useful indicator of the fit of a given model owing to its sensitivity to sample size, for example (eg, as noted by Kelloway [48]), it can be useful in terms of model comparison [47]. Thus, a change in thechi-square value was used to test the improvement of a given model over a previous model. Other fit indices included the CFI, normed fit index (NFI), NNFI, and RMSEA. Akaike’s information criterion (AIC) was used as another indicator of comparative fit, where a lower AIC indicated a relatively better model fit. As noted, identifying precise cutoffs for model fit is probably unrealistic, given the behavior of fit statistics under varying conditions [20,49], as is shown by others [46]. Thus, we interpreted fit by consulting multiple indices and used a cutoff based upon *adequate fit*. Specifically, a fit of ≤ .08 was deemed adequate for the RMSEA and Standardized Root Mean Square Residual, and a fit of ≥ .90 was deemed adequate for the CFI, NFI, and NNFI [49]. Measurement invariance among men and women was tested using standard procedures [50], for example, using IBM AMOS 24 [43]. As we were only interested in testing whether or not the proposed CFA model held good for men and women, we tested configural, metric scale, and residual forms of invariance [49]. Finally, the relationships between the PRFQ-18 subscales and the subscales of the MOS-SSS, PSOC, PSS, and PCS data were investigated using bivariate correlations in IBM SPSS Statistics 23. RStudio (using R 3.3.2) was used to calculate ordinal alpha and omega forms of scale reliability [51] and to test for structural invariance.

## Results

### Sample Description

A total of 344 Canadian adult parents (aged 20 to 60 years) with at least 1 child between the age of 0 and 12 years were randomly sampled through SurveyMonkey’s *Survey Audience*. After screening for eligibility, 317 participants completed the PRFQ-18 and 306 participants (120 male and 186 female) completed all study measures. Participant demographics of our diverse sample are reported in Table 1. Of the 306 participants who completed all study measures, most parents had 1 (n=106) or 2 (n=132) children in the home with 68 parents having more than 2 children in the home.

**Table 1:** Demographics of the sample population.

Variable		Males (n=120), n (%)	Females (n=186), n (%)	Total (n=306), N (%)
**Age (years)**				
	20-29	15 (12.5)	33 (17.7)	48 (15.7)
	30-39	60 (50.0)	91 (48.9)	151 (49.3)
	40-49	36 (30.0)	55 (29.6)	91 (29.7)
	50-60	9 (7.5)	7 (3.8)	16 (5.2)
**Age of child(ren) in household (years)**				
	0-3	36 (30.0)	83 (44.6)	119 (38.9)
	4-6	52 (43.3)	64 (34.4)	116 (37.9)
	7-9	49 (40.8)	64 (34.4)	113 (36.9)
	10-12	37 (30.8)	65 (34.9)	102 (33.3)
	13 and older	23 (19.2)	45 (24.2)	68 (22.2)
**Children in household**				
	Biological	118 (38.6)	184 (60.1)	302 (98.7)
	Step	37 (12.1)	52 (17.0)	89 (29.1)
	Foster	32 (10.5)	51 (16.7)	83 (27.1)
	Adopted	32 (10.5)	51 (16.7)	83 (27.1)
**Education**				
	Less than high school degree	4 (3.3)	6 (3.2)	10 (3.2)
	High school degree or equivalent	20 (16.7)	26 (14.0)	46 (15.0)
	Some college but no degree	11 (9.2)	29 (15.6)	40 (13.1)
	Technical degree or diploma	26 (21.7)	44 (23.7)	70 (22.9)
	Bachelor’s degree	44 (36.7)	59 (31.7)	103 (33.7)
	Graduate degree	15 (12.5)	22 (11.8)	37 (12.1)
**Household income (Can $)**				
	0-24,999	6 (5.0)	14 (7.5)	20 (6.5)
	25,000-74,999	47 (39.1)	75 (40.4)	122 (39.9)
	75,000-124,999	44 (36.7)	62 (33.3)	106 (49.0)
	125,000-199,999	18 (15.1)	20 (10.8)	38 (20.3)
	200,000 and above	2 (1.7)	1 (.5)	3 (1.0)
	Prefer not to answer	3 (2.5)	14 (7.5)	17 (5.56)
**Relationship status**				
	Married	90 (75.0)	131 (70.4)	221 (72.2)
	Widowed	1 (0.8)	1 (0.5)	2 (1.0)
	Divorced	7 (5.8)	6 (3.2)	13 (4.2)
	Separated	3 (2.5)	10 (5.4)	13 (4.2)
	Common law	12 (10.0)	21 (11.3)	33 (10.8)
	Single, never married	6 (5.0)	16 (8.6)	22 (7.2)
	Open relationship	1 (0.8)	1 (0.5)	2 (1.0)
**Residence (province)**				
	Western Canada	32 (26.7)	71 (38.2)	103 (33.6)
	Eastern Canada	86 (71.1)	115 (61.9)	201 (65.7)
**Race/ethnicity**				
	Asian and/or Pacific Islander	17 (14.0)	34 (18.2)	50 (14.7)
	African	2 (1.7)	0 (0.0)	2 (0.6)
	Black	1 (0.8)	1 (0.5)	2 (0.6)
	Canadian	8 (6.7)	14 (7.5)	22 (5.8)
	Caucasian	80 (66.7)	117 (62.9)	198 (58.2)
	European	3 (2.5)	1 (0.5)	4 (1.2)
	First Nations	0 (0.0)	4 (2.2)	4 (1.2)
	Latin	1 (0.8)	2 (1.1)	3 (0.9)
	Middle Eastern	2 (1.6)	2 (1.0)	4 (1.2)
	Mixed	3 (2.5)	3 (1.6)	7 (2.0)
	Prefer not to say	3 (2.5)	9 (4.8)	12 (3.5)

### Parent Reflective Functioning Questionnaire-18 Factor Structure

**Figure 1:**
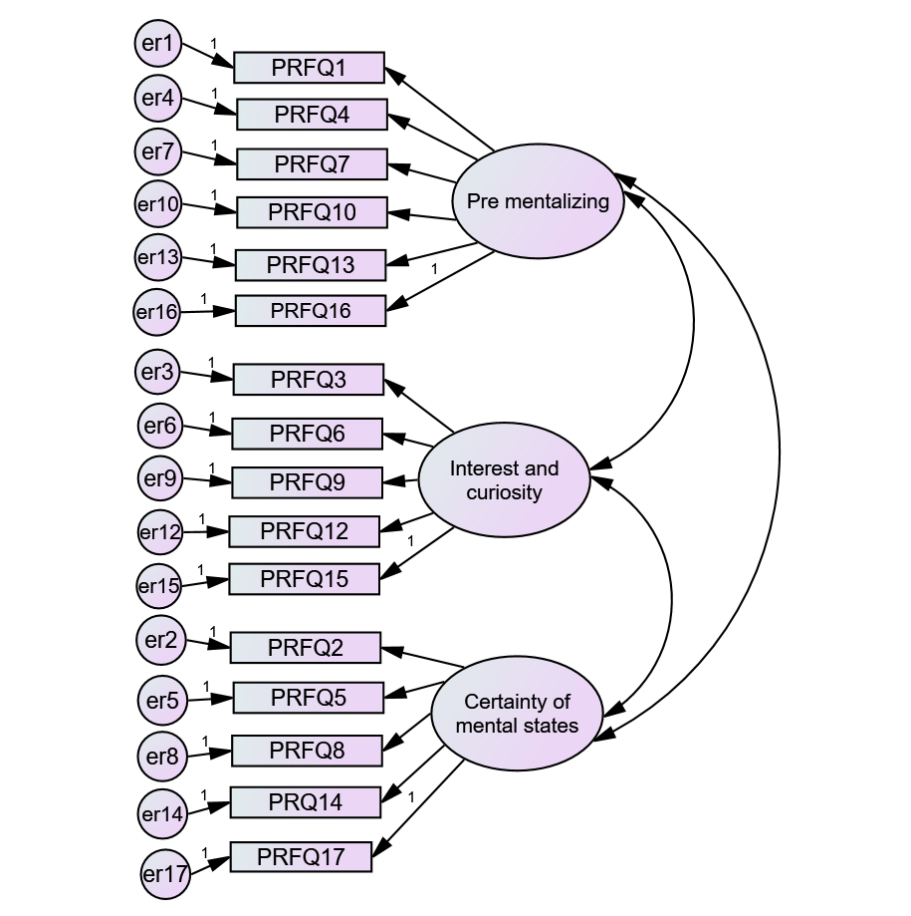
The Parent Reflective Functioning Questionnaire (PRFQ) model without items 11 and 18.

Factor structure analysis of the PRFQ-18 resulted in examining 4 different CFA models (see Table 2) utilizing 317 participants. The initial CFA model testing of the hypothesized 3-factor structure of the PRFQ-18 (PM, CMS, and IC) resulted in a reasonably poor model fit, as shown in Table 2. Results of the initial CFA indicated that Item 11 was not significantly (*P*=.10) contributing to the PRFQ-18 measure. To decipher whether or not the model fit could improve with the removal of Item 11, a second CFA was conducted. Results in Model 2 found improvements in the model fit in terms of the fit statistics reported in Table 2, and the chi-square difference test showed that the change in the chi-square value from Model 1 to 2 was significant (chi-square difference=138, *P*<.001). However, Model 2 showed a low standardized regression weight for Item 18 (.27). To attempt enhancing the model fit even further, Item 18 was removed in Model 3. After testing Model 3, results identified a more respectable model fit (see Table 2, chi-square difference test=.210 *P*<.001) and modification indices suggested a better model fit by adding a covariance between error terms 6 and 9. Adding a correlated error term between the errors for items 6 and 9 further improved the model fit as seen in Table 2 (Model 4, chi-square difference test=52; *P*<.001). Model 4 resulted in a negative correlation between PM and IC (−.26) and positive correlations between CMS and IC (.36) and between CMS and PM (.37), suggesting that the PRFQ-18 measures 3 relatively independent characteristics of PRF (see Figure 1). On the basis of this model, internal consistencies for the PM, IC, and CMS subscales were alpha=.91, .88, and .88, and omega=.91, .92, and .95, respectively.

A test of measurement variance showed strong support for invariance between men and women. The test for configural invariance showed that the same factor model applied for both men and women (χ^2^_200_=435.60; *P*<.001; RMSEA=.062; 90% CI 0.054 to .070; CFI=.91; AIC=579.56). Measurement/metric invariance was also supported as seen in a change in chi-square, χ^2^_13_=8.42, *P*=.82, and a small change in CFI of .001. Compared with the measurement invariance, structural invariance and then residual invariance showed a change in CFI of less than .001 each. These results show strong support for invariance between men and women suggesting the tool functions similarly among men and women.

**Table 2:** Fit statistics for confirmatory factor analysis models.

Model	Chi-square (*df*)	Chi-square*/df*	Root mean square error of approximation (90% CI)	Comparative fit index	Normed fit index	Non-normed fit index (Tucker Lewis Index)	Akaike Information Criterion	Standardized Root Mean Square Residual
Model 1^a^	703 (132)	5.33	0.117 (0.109-0.126)	.81	.78	.78	781	.144
Model 2	565 (116)	4.87	0.111 (0.102-0.120)	.84	.81	.82	639	.137
Model 3	355 (101)	3.52	0.089 (0.079-0.099)	.91	.87	.89	425	.081
Model 4	303 (100)	3.03	0.80 (0.070-0.091)	.92	.89	.91	375	.077

^a^Initial model contains all 18 items: Model 2 has Item 11 removed, Model 3 has Items 11 and 18 removed, and Model 4 adds a correlated error term to Model 3.

### Intercorrelations Among Measures

Following the CFA, we proceeded to examine the intercorrelations among study variables with the revised version of the PRFQ that does not include items 11 and 18. Complete data for these analyses were available for 306 participants.

#### Relationships With Parenting Sense of Competence

Similar to preliminary findings [13], the PSOC demonstrated a range of correlation results across the PRFQ-18 subscales (see Multimedia Appendix 1). Negative correlations were found between PM and satisfaction (r_males_=−.65; r_females_=−.50) and efficacy for females (r_females_=−.14). This suggests that parents with an inability to hold their child’s mental state also have a lower sense of satisfaction with their parenting and a lower sense of efficacy for mothers. As expected, the IC subscale was positively correlated with efficacy (r_males_=.44; r_females_=.28) but not with satisfaction. These results indicate that parents with relatively higher levels of interest and curiosity in their child’s mental state also have relatively higher levels of satisfaction as a parent and perceptions of parental competence. Finally, the CMS subscale had positive correlations with both satisfaction in men (r_males_=.21) and efficacy for both men and women (r_males_=.43; r_females_= .32). This shows that parents who are more certain of their child’s mental states report more efficacy for the parenting role. Furthermore, for fathers, more certainty around mental states was associated with more satisfaction around being a parent.

#### Relationships With Stress

Consistent with preliminary findings [13], both the IC (r_males_=−.17; r_females_=− .09) and CMS (r_males_=−.16; r_females_=−.13) subscales were negatively correlated with stress, but these were not significant. On the contrary, PM was positively correlated with stress (r_males_=.30; r_females_=.27), that is, those reporting higher levels of stress had a relatively higher inability to hold their child’s mental state, as expected.

#### Relationships With Social Support

As expected, correlation results from the MOS-SSS subscales varied across the PRFQ-18 subscales. The PM subscale was negatively correlated to tangible support (r_males_=−.27; r_females_=−.13), emotional information support (r_males_=−.21; r_females_=−.24), affectionate support (r_males_=−.24; r_females_=−.28), and positive interaction (r_males_=−.23; r_females_=−.23) subscales. That is, those reporting less social support tended to report higher levels of prementalization. The IC subscale was positively correlated to tangible support (r_males_=.27; r_females_=.17), emotional information support (r_males_=.19; r_females_= .19), affectionate support (r_males_=.28; r_females_= .22), and positive interaction (r_males_=.22; r_females_=.19) subscales. Thus, those with relatively more social support reported higher levels of interest and curiosity in their child’s state of mind. Finally, the CMS subscale also had positive but nonsignificant correlations with social support subscales.

#### Relationships With Parental Coping

In line with our hypothesis, the PCS had positive correlations with IC (r_males_=.37) and CMS (r_males_=.33; r_females_=.14) and negative correlations among PM (r_males_=−.21; r_females_=−.28; see Multimedia Appendix 1). In other words, those with a relatively high ability to cope with parenting also display better levels of mentalization than those with a relatively lower ability to cope.

#### Relationships Between Mothers and Fathers

Interestingly, key findings identified a variety of commonalities between mothers and fathers. Both mothers and fathers had negative correlations among PM (r_males_=−.65; r_females_=−.50) and satisfaction. In addition, both mothers and fathers had positive correlations between both IC (r_males_=.44; r_females_= .28) and CMS (r_males_=.43; r_females_= .32) with efficacy. On the contrary, mothers had negative correlations (r_females_=−.27) and fathers had nonsignificant correlations between PM and efficacy. In correspondence to preliminary findings [13], we found positive correlations among PM (r_males_=.30; r_females_=.27) and parental stress for both mothers and fathers [13], that is, a higher perceived stressful situation was associated with low mentalization in parents. In addition, both mothers and fathers had negative correlations between PM and social support subtypes. The exception was tangible support which was not related to prementalization in women. Overall, parents with more social support tended to have higher levels of RF. Finally, results showed negative correlations within PM and parental coping but only positive links with IC in fathers. Overall, these findings suggest that parents with high RF capabilities are better able to cope with parenting but that the type of RF related to coping might vary between mothers and fathers.

## Discussion

### Overview

The PRFQ is a brief self-report instrument designed to assess parent RF capacities [13]. Support for the validity of the PRFQ-18 has been presented previously in mothers and fathers [13]. To date, we have been unable to find any independent studies examining the factor structure and testing the discriminant and construct validity of the PRFQ-18. This study intended to further test the factor structure by means of a CFA and to expand the discriminant and construct validity of the PRFQ-18 by exploring relationships between this measure and the MOS-SSS, PCOS, PCS, and the PSS in a Canadian sample (n=306) of parents of children aged 0 to 12 years.

Results of this study extend and replicate earlier findings of the PRFQ-18 factor structure. Specifically, the CFA supported a 3-factor structure capturing key characteristics of RF: (1) prementalizing, (2) interest and curiosity, and (3) certainty of mental states. In addition, our results suggest items 11 and 18 may not be contributing to the measurement of PRFQ-18. Interestingly, items 11 and 18 are negatively worded and when removed, improved the PRFQ-18 model fit changes from poor to acceptable, as indicated in the fit measures. This may not be surprising as a mix of negative and positively worded items has the potential to cause problems as positively and negatively worded items may not be measuring the same underlying trait [52]. Taking this into consideration, it seems the removal of items 11 and 18 may be appropriate when using the PRFQ-18 in the future. Weak loadings for items 11 and 18 were also reported in preliminary studies [13]. However, given the early state of the PRFQ-18, 2 items in the PRFQ-18 were removed [13]. Our results suggest that it may be prudent to conduct further research examining the model fit of the 16-item measure reported here.

### Validity Evidence

In terms of predictive and discriminant validity, the PRFQ-18 subscales were correlated with the MOS-SSS, PCOS, PCS, and PSS subscales in the expected directions, that is, the PRFQ-18 subscales were generally found to correlate with perceived social support, parental competence, and parent’s perceptions of coping abilities and stressful situations in the right direction. Furthermore, the intercorrelations among PRFQ-18 subscales were low-to-moderately correlated, supporting a relative distinction among these subtypes adding support to the notion of 3 separate PRF concepts.

In terms of the PRFQ-18 construct validity support, prementalizing had negative correlations with coping, efficacy, and forms of social support. This result is similar to findings that individuals capable of perceiving themselves as parents and their relationships with their child will seek out social support [22]. In addition, PM was seen to have a positive relationship with satisfaction and perceived stress. These results are comparable to findings that identify parents with low RF as being unable to imagine the type of support they would need and if it would be available [23].

### Theoretical Explanations

Interestingly, our results identified IC as positively correlated with parental coping, emotional information support, affectionate support, positive interaction, tangible support, and efficacy. These outcomes match findings that suggest parents with greater levels of parenting coping abilities and an awareness of stress display higher levels of RF, influencing how they feel about their parenting capabilities [23]. On the contrary, IC was negatively correlated to perceived stress and satisfaction, that is, a parent’s awareness and curiosity of a child’s mental state is relatively low among those reporting higher levels of stress and dissatisfaction with their parenting. These results are consistent with research and theory arguing that stress can impair one’s mentalization [53]. This consistency adds construct validity support to the PRFQ measure.

Furthermore, CMS was positively correlated with satisfaction, efficacy, social support types, and coping. These results confirm that a parent’s perception that their thoughts about their child’s mental states are accurate contributes to their feelings of satisfaction, parental competence, and ability to manage and cope with parenting. In contrast, CMS was found to have nonsignificant negative associations with perceptions of stress. This suggests that stress may have less of an impact on this form of RF. This result requires further study, as it may speak to how specific forms of RF operate under different circumstances. In sum, our results show that PRFQ subscales are generally related to other social cognitive variables as one would expect based on the literature, adding support for the construct validity of the measure. Although the PRFQ-18 is a fairly new measure within the field of psychology [54], our results are consistent with those reported in preliminary studies [13].

Finally, we identified both similarities and differences among mothers and fathers in terms of PRFQ correlates. In general, both mothers and fathers with high RF had lower levels of parental stress and more social support, satisfaction, efficacy, and better coping. However, some differences in terms of how RF operates in terms of coping and competence among men and women were noted here. Specifically, men who reported good coping with parenting reported more interest and curiosity in their child’s mental state, more certainty around understanding their child’s mental state, and better mentalization. Another notable difference was the relationship between parental efficacy and PM. Women with more parental efficacy had lower levels of PM and, thus, better mentalization. In contrast, PM was unrelated to a father’s efficacy. For women, good coping was primarily related to better mentalization, as seen in lower PM scores. However, for fathers, satisfaction with parenting was strongly negatively correlated with PM, showing that fathers with a great deal of satisfaction with being a parent also have a better ability to mentalize.

### Limitations and Future Directions

Advantages of self-report measures include time efficiency, cost-effectiveness, and ease of administration. Self-report measures are reported to be valid measures in examining cognitive constructs, emotions, and moods [55]. However, one issue concerning self-report measures is the level of insight required from an individual [55]. Other cautions against self-report measures include potential inaccuracy in participants’ answers and various response styles influencing results [56]. To further support the validity of the PRFQ-18 and to allay concerns about the PRFQ-18 being a self-report measure, future research should consider including a gold standard measure such as the PDI to compare these results observed here to uncover if the PRFQ-18 can offer insights similar to the current gold standard measures. In addition, this research would provide more evidence as to the reliability and validity of the PRFQ-18.

A limitation of this study is the reliance on a preexisting survey panel. Whereas this method allows for easy access to a wide cross-section of Canadians, the survey panel may differ in unknown and important ways from a true random sample of Canadians. If future research could replicate these findings in a random sample of Canadians recruited through other means, we would have increased confidence in the findings in this study.

Ideally, an independent sample should be used as a follow-up to our CFA to determine the extent to which PRFQ-18 subscales are related to theoretically meaningful constructs. However, a follow-up study should attempt to replicate our findings to see if the removal of items 11 and 18 is supported in other samples. Considering the preliminary studies had similar findings to this study [13], we would expect this result to hold. In this study of parents with children aged 0 to 12 years, we found evidence supporting the construct validity of a revised PRFQ; however, it is possible that the PRFQ might perform differently between younger and older children [13]. This is a question for future research to examine in an independent sample. Finally, variances among mothers and fathers suggest further exploration between mothers, fathers, RF, and cognitive variables is needed. That is, our results suggest that RF subtypes may be differentially influenced by the social and psychological parenting context.

## Appendix

Multimedia Appendix 1 Summary of intercorrelations, means and stand deviations for scores on the Parent Reflective Functioning Questionnaire -18, Medical Outcome Study Social Support Survey, Parenting Sense of Competence Scale, Perceived Stress Scale, Parent Coping Scale in males and females.

## Abbreviations

CFA: confirmatory factor analysis
CFI: comparative fit index
CMS: certainty about mental states
IC: interest and curiosity in mental states
MOS-SSS: Medical Outcome Study Social Support Survey
NFI: normed fit index
NNFI: non-normed fit index
PCS: Parent Coping Scale
PDI: Parent Development Interview
PM: prementalizing modes
PRF: parental reflective functioning
PRFQ: Parent Reflective Functioning Questionnaire
PSOC: Parenting Sense of Competence Scale
PSS: Perceived Stress Scale
RF: reflective functioning
RMSEA: root mean square error of approximation
SSP: Strange Situation Procedure
