# Supplementary material for: Advancing Optimal Development in Children: Examining the Construct Validity of a Parent Reflective Functioning Questionnaire
Source: JMIR Pediatr Parent. 2019 May 9;2(1):e11561. doi: 10.2196/11561 (PMC6716429; doi:10.2196/11561)
Supplement: Multimedia Appendix 1 [file pediatrics_v2i1e11561_app1.pdf]

Appendix 1. Summary of Intercorrelations, Means and Stand Deviations for Scores on the PRFQ-18, MOS-SSS, PSOC, PSS, PCS in Males and Females

Measure		1	2	3	4	5	6	7	8	9	10	11	12	13	14	*M*	*SD*
**1.**	**PRFQ**	-	.62**	.40**	.80**	.00	.06	-.03	-.03	.01	.37**	.29**	.15*	.05	-.04	4.05	.80
2.	PM	.73**	-	-.27**	.25**	-.24**	-.13	-.24**	-.28**	-.23**	.23**	.50**	-.27**	.27**	-.28**	2.53	1.42
3.	IC	.63**	.05	-	.21**	.21**	.17*	.19**	.22**	.19**	.16*	-.06	.28**	-.09	.11	5.43	1.06
4.	CMS	.84**	.45**	.43**	-	.09	.10	.06	.06	.10	.28**	.04	.32**	-.13	.15*	4.20	1.35
**5.**	**MOS-SSS**	.01	-.26**	.26**	.09	-	.85**	.94**	.91**	.94**	-.01	-.35**	.39**	.47**	.34**	3.70	.94
6.	Tangible	-.02	-.27**	.27**	.10	.85**	-	.70**	.65**	.71**	-.00	-.27**	.31**	-.38**	.24**	3.39	1.07
7.	EI	.02	-.21*	.19*	.06	.94**	.70**	-	.82**	.88**	-.00	-.32**	.36**	-.45**	.30**	3.73	1.08
8.	Affectionate	.03	-.24**	.28**	.06	.91**	.65**	.82**	-	.83**	-.04	-.35**	.35**	-.42**	.32**	3.93	1.00
9.	PI	.01	-.23*	.22*	.10	.94**	.71**	.88**	.83**	-	-.00	.35**	.40**	-.47**	.36**	3.73	.99

10.	**PSOC**	.52**	.51**	.17**	.28**	-.01	-.04	-.04	-.04	-.00	-	.68**	.54**	.17*	-.13	3.65	.56
11.	Satisfaction	.39**	.65**	-.09	.04	-.35**	-.27**	-.32**	-.35**	-.35**	.68**	-	-.26**	.56**	-.45**	3.07	.97
12.	Efficacy	.40**	.10	.35**	.32**	.39**	.31**	.36**	.35**	.40**	.54**	-.26**	-	-.42**	.34**	4.24	.85
13.	**PSS Total**	.02	.30**	-.17	-.13	-.47**	-.38**	-.45**	-.42**	-.47**	.17*	.56**	-.42**	-	-.49**	1.56	.65
14.	**PCS Total**	.18*	-.21*	.37**	.15*	.34**	.24**	.30**	.32**	.36**	-.13	-.45**	.34**	-.49**	-	3.54	.89
	M	4.11	3.12	5.03	4.18	3.61	3.50	3.58	3.74	3.62	3.72	3.33	4.11	1.59	3.42	-	-
	SD	.91	1.44	1.15	1.15	.90	1.04	.97	.98	.96	.73	1.04	.88	.66	.90	-	-

Note: Intercorrelations for females (n= 186) are presented above the diagonal and intercorrelations for males (n= 120) are presented below the diagonal. Means and standard deviations for females are presented in the vertical columns and means and standard deviations for males are presented in the horizontal rows. PRFQ-18 = Parent Reflective Functioning Questionnaire; PM= Pre-mentalizing; IC=Interest & curiosity; CMS= Certainty of mental states; MOS-SSS= Medical outcome study social support survey; EI= Emotional-information; PI= Positive interaction; PSOC= Parenting sense of competence scale; PSS = Perceived stress scale; PCS= Parenting coping scale. ** $P < 0.01$ (2-tailed); * $P < 0.05$ (2-tailed)
